# Supplementary material for: Amazon windthrow disturbances are likely to increase with storm frequency under global warming
Source: Nat Commun. 2023 Jan 6;14:101. doi: 10.1038/s41467-022-35570-1 (PMC9822931; doi:10.1038/s41467-022-35570-1)
Supplement: Supplementary file 1 — Supplementary Information [file 41467_2022_35570_MOESM1_ESM.pdf]

Supplementary Information For

Amazon windthrow disturbances are likely to increase with storm frequency under global warming

Yanlei Feng[1*], Robinson I. Negron-Juarez[2], David M. Romps[2,3], Jeffrey Q. Chambers[1,2]

[1]Department of Geography, University of California, Berkeley, California, USA
[2]Climate and Ecosystem Sciences Division, Lawrence Berkeley National Laboratory, Berkeley, California, USA
[3]Department of Earth and Planetary Science, University of California, Berkeley, California, USA

*Corresponding author. Email: ylfeng@berkeley.edu

This file includes:
Supplementary figures (Fig. 1-8)
Supplementary tables (Table 1-3)
Supplementary references 1-11

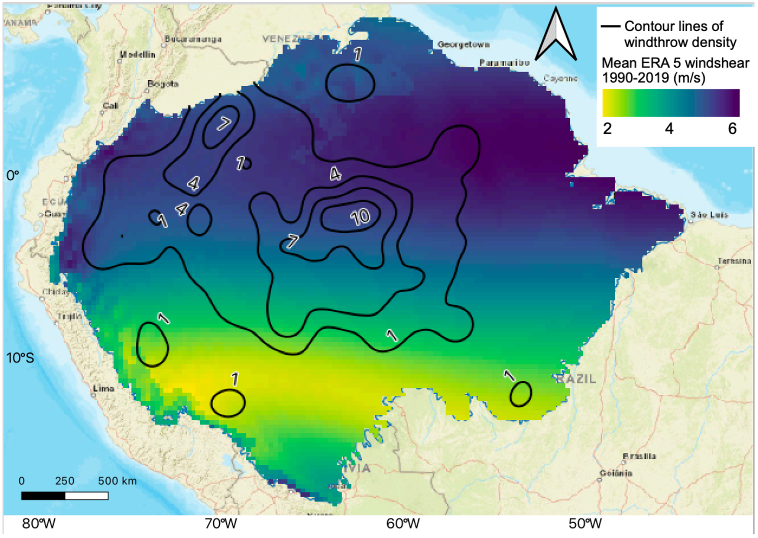

Supplementary Fig. 1 Mean monthly windshear over 30 years (1990-2019). We find no correspondence between climatological wind shear patterns and windthrow density. Wind shear was calculated as the difference between the horizontal wind vector near the surface and 6km above the surface. The calculation of windshear in this study followed Seeley and Romps 2015[1] and used ERA5 monthly averaged V-component of wind and U-component of wind on surface pressure and 100 mbar.

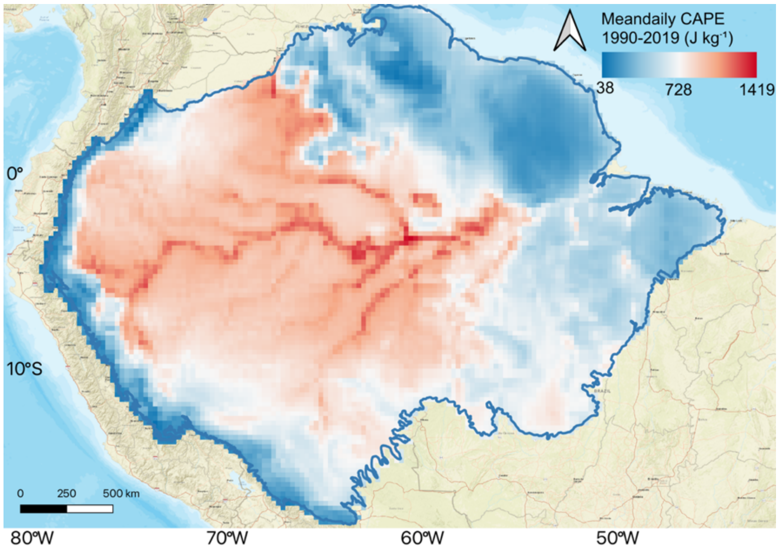

Supplementary Fig. 2 Mean daily convective available potential energy (CAPE) over 30 years. The map was calculated using an average of daily (24-hour) CAPE datasets over 1990-2019.

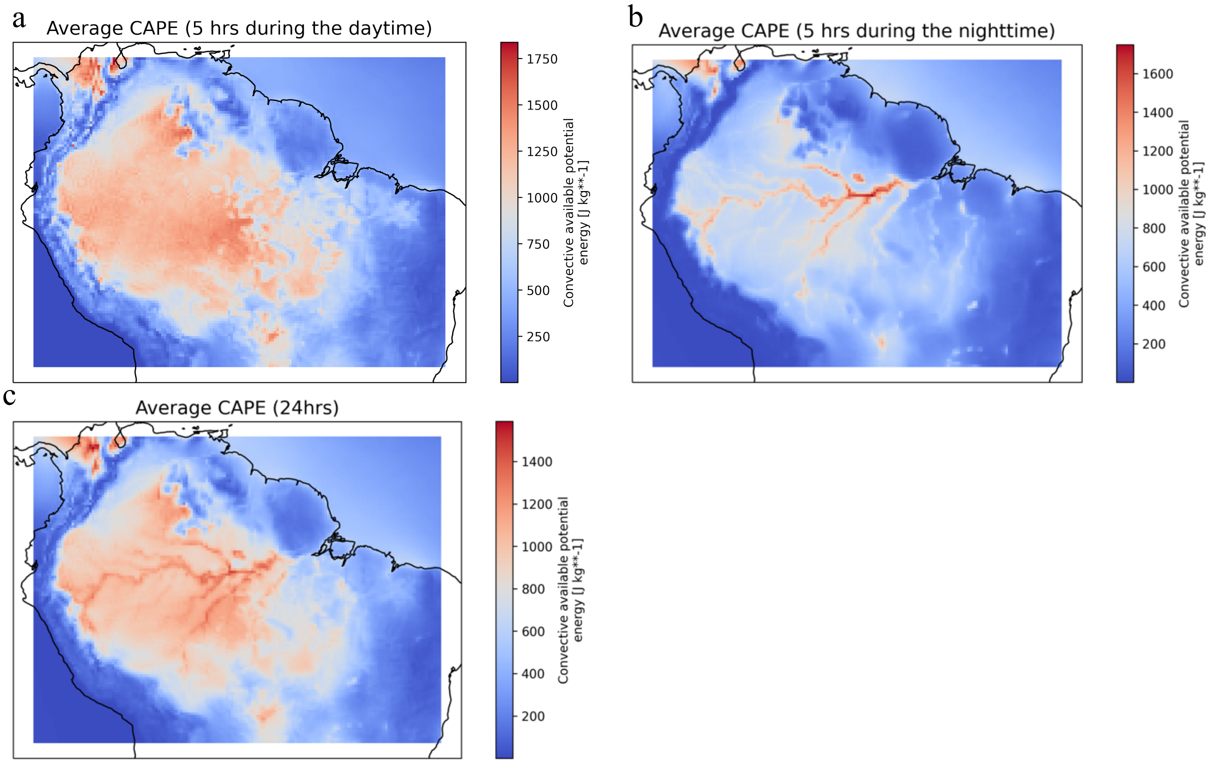

Supplementary Fig. 3 A comparison of mean hourly convective available potential energy (CAPE) using different calculations. Mean hourly CAPE over (a) 13:00-17:00 local time, (b) 00:00-04:00 local time, (c) 24 hours during 1990-1994. Mean daily CAPE has high values over rivers due to nighttime river intensification.

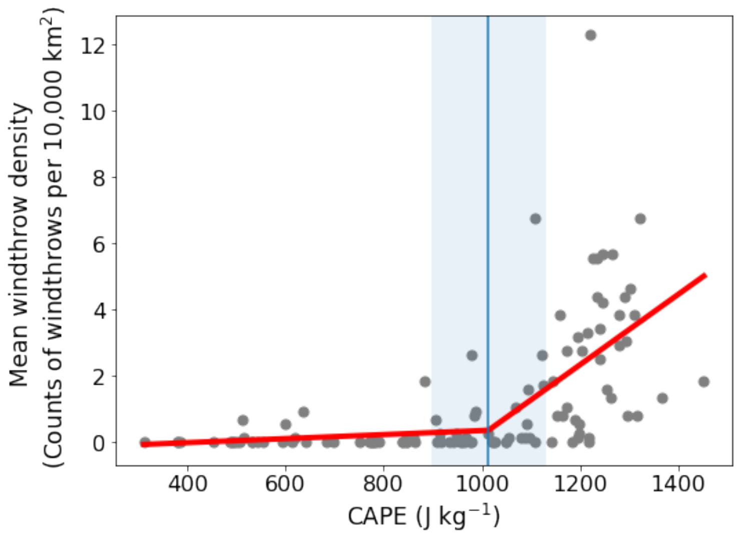

Supplementary Fig. 4 A segmented regression using aggregated mean afternoon convective available potential energy (CAPE) and windthrow density datasets from Fig. 1b and 1d shows that a threshold exists at CAPE of 1014 J kg^{-1} ($r^2 = 0.39$, $p < 0.05$). The confidence interval of the threshold is 898- 1130 J kg^{-1}.

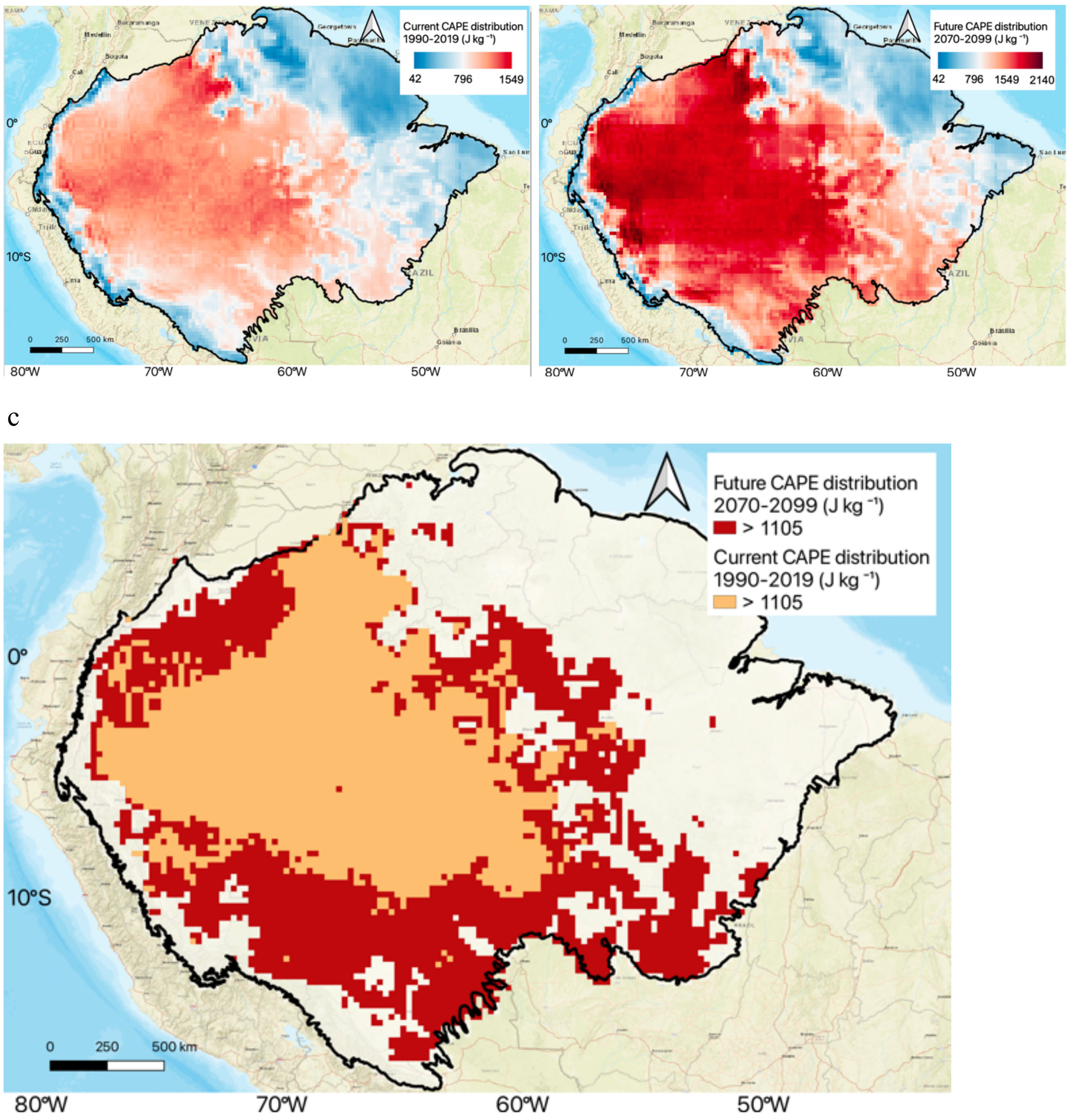

Supplementary Fig. 5 The comparison of the spatial pattern of current and future convective available potential energy (CAPE) and the increase in regions with high CAPE. (a) Current and (b) future spatial pattern of ERA 5 mean CAPE values across the Amazon region using the same color palette; (c) The spatial pattern of the increase in area of high CAPE (>1105 J kg^{-1}), orange pixels represent current CAPE higher than 1105 J/kg while red pixels represent future CAPE higher than 1105 J/kg. Current CAPE was calculated as the mean of ERA 5 hourly CAPE dataset from 1990-2019, and future mean CAPE was calculated using increase percentage in CMIP 6 CAPE from the past 30 years (1990-2019) to the last 30 years in the century (2070-2099) and then applied the increase percentage to ERA 5 CAPE afternoon mean.

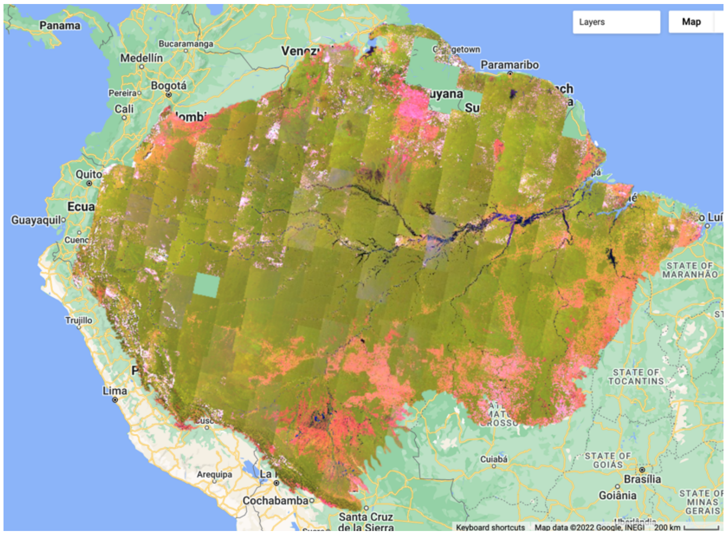

Supplementary Fig. 6 Landsat scenes used to identify 1012 windthrows. Map showing Landsat images covering the Amazon used in this study.

a

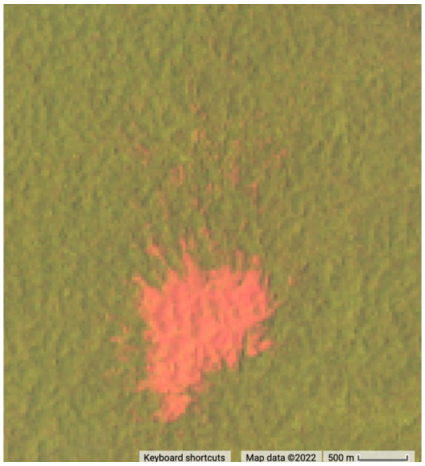

"New" windthrow centered at -74.25634 longitude, -3.75625 latitude, displayed in Landsat composite from 2018-2019. The windthrow occurred in 07/30/2017.

b

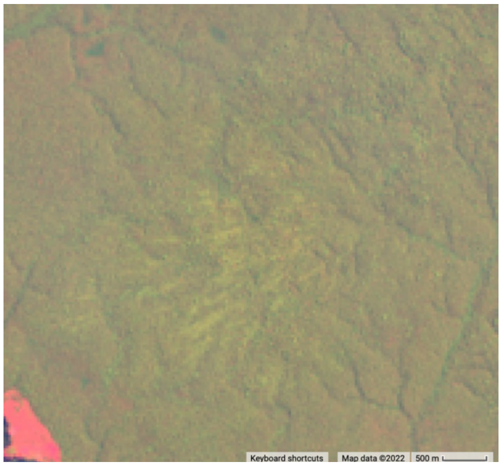

"Old" windthrow centered at -59.12147 longitude, -2.81343 latitude, displayed in Landsat composite from 2018-2019. The windthrow occurred in 10/16/2000.

c

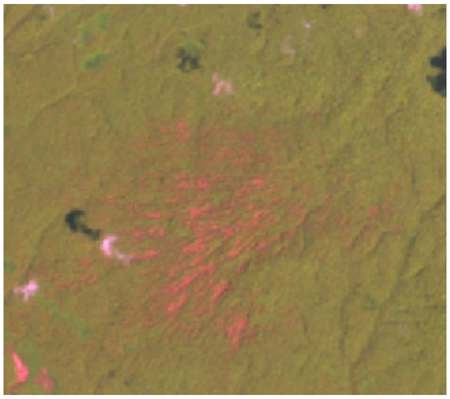

The same “old” windthrow as Supplementary Fig. 7 (b) displayed in Landsat 5 image from 11/29/2000. The shape and color of the windthrow is more visible and this image can be used to validate the existence of this “old” windthrow.

Supplementary Fig. 7 “New” and “old” windthrow displayed in Landsat composite in false color (red: shortwave infrared band, green: near infrared band, blue: red band). (a) “New” Windthrow that occurred within 1 years of identification displayed in satellite images from 2018-2019. The reddish color indicated that the windthrow was relatively fresh with lots of dead trees with high reflectance in shortwave infrared band; (b) “Old” windthrow that occurred nearly 20 years ago from identification displayed in satellite images from 2018-2019; (c) “Old” windthrow that occurred nearly 20 years ago from identification displayed in satellite images from 2000.

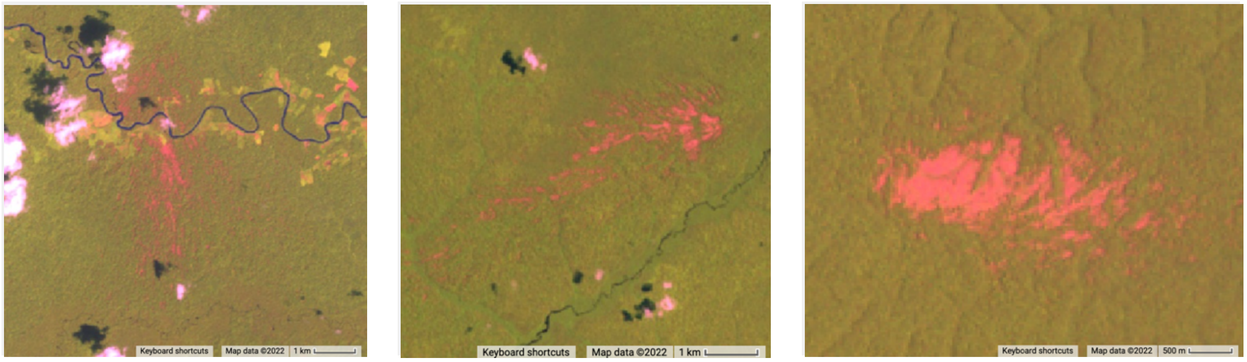

(-75.61695, 0.36378) (-65.28033, -1.24827) (-61.20923, -2.21437)

Supplementary Fig. 8 Three windthrow events displayed in the reddish color on Landsat 5 TOA images with false color visualization (red: shortwave infrared band, green: near infrared band, blue: red band). Each windthrow displayed shows a clear fan shape diverging from a head with scattered small disturbance pixels spreading at the tail, indicating wind direction from windthrow head to tail. This distinctive shape helps authors determine the windthrow event and separate it from other type of disturbances. The locations of windthrows are displayed underneath each figure with longitude and latitude.

Supplementary Table 1 The Earth system models (ESMs) included in this study. Resolution is indicated in terms of (longitude pixels) × (latitude pixels).

ESM	Institute	Resolution
BCC, BCC-CSM2-MR[2]	Beijing Climate Center	27*54
CCCma, CanESM5[3]	Canadian Centre for Climate Modeling and Analysis	11*22
CAS, FGOALS-g3[4]	Institute of Atmospheric Physics, Tsinghua University	15*31
MIROC, MIROC6[5]	Japan Agency for Marine-Earth Science and Technology	21*43
MIROC, MIROC-ES2L[6]	Japan Agency for Marine-Earth Science and Technology	11*22
KIOST, KIOST-ESM[7]	Korea Institute of Ocean Science and Technology	32*16
MRI, MRI-ESM2-0[8]	Meteorological Research Institute	27*54
CSIRO-ARCCSS, ACCESS-CM2[9]	Commonwealth Scientific and Industrial Research Organisation (CSIRO) and ARCCSS (Australian Research Council Centre of Excellence for Climate System Science	24*32
MPI-M, MPI-ESM1-2[10]	Max Planck Institute	16*33
CNRM-CERFACS, CNRM-CM6-1[11]	National Center for Meteorological Research, Météo-France and CNRS laboratory	21*43

Supplementary Table 2 The occurrence year of 125 windthrows

Location (longitude, latitude)	The year of occurrence
-75.31885, -3.42043	2002
-75.1687, -3.46831	2010-2011
-75.28461, -2.93377	2011
-72.95713, -1.69526	2016
-72.30887, -2.04454	2018
-71.07156, -4.56275	2008
-69.5837, -3.9108	2004
-69.9149, -0.399	2009
-70.146, -0.4903	2009
-69.2315, -6.072	2000
-67.00852, -3.80105	2000
-73.36516, -1.88098	2002
-75.5326, 0.0397	2005
-74.1691, -0.3449	2004
-71.49935, -4.04714	2008
-70.74026, -3.25694	2014
-70.75365, -3.29568	2012
-71.7809, -3.0639	2004

-74.4379, -3.8458	2011
-70.6677, -2.2852	2018
-71.1278, -1.1604	2017
-70.7599, -3.2992	2013-2014
-70.7372, -3.2618	2014-2015
-75.21286, -3.01703	2010
-73.7513, -10.3512	2008
-68.4226, -7.0308	2007
-65.9308, -6.4306	2011-2013
-64.1628, -6.38	2011-2013
-65.8689, -5.8894	2005-2006
-71.5945, -2.4083	2004
-70.4345, -2.7951	2011-2013
-70.9944, -4.9434	2008
-71.86433, -4.25689	2011-2013
-71.49783, -4.04596	2008
-70.445, -2.8011	2011-2014
-74.25175, -3.75808	2017
-71.16435, -1.76144	2017
-71.29465, -2.12257	2014-2015
-70.60843, -2.02333	2007-2008
-70.10827, -2.46262	2010
-70.9078, -2.68942	2010
-68.9531, -1.135	2010
-70.5549, -2.6825	2002-2003
-68.09293, -2.08852	2013
-68.5063, -2.6105	2006-2007
-68.6805, -4.6648	2017
-69.7147, -4.761	2017-2018
-70.4033, -5.6958	2007-2008
-69.586, -6.8061	2013-2014
-65.27829, -1.24835	1998
-61.91331, -2.74979	2003
-61.12781, -2.9963	2017
-62.3112, -6.80019	2016
-61.49781, -1.12337	2019
-60.0886, -3.8451	2005

-62.9612, -2.5185	2018
-59.1245, -2.8039	2000
-61.3818, -2.641	1992-1993
-61.5312, -0.5627	before 1998
-62.3731, -0.5505	2002
-62.60639, -0.31402	2006
-65.57273, -3.55288	2006
-65.57446, -3.55848	2006
-60.27267, -2.56408	2004-2005
-60.17982, -2.26191	1996
-57.91189, -1.85828	2008
-66.27618, -2.81099	2006
-68.2653, -2.8582	2003
-67.00124, -4.04004	2000
-65.99758, -4.05031	1996
-63.9083, -2.1432	1994
-63.8099, -2.2193	1993
-63.36, -1.6083	1997
-64.1962, -2.9454	2009
-66.7111, -1.8356	2018
-64.5004, -2.701	2015
-67.8126, -1.9156	2011
-67.5478, -1.9685	1989
-66.77318, -3.50401	before 1986
-66.94516, -3.42641	2004
-67.00767, -3.4189	before 1997
-65.3092, -3.6728	2008
-62.4458, -3.4435	2004
-62.68488, -3.18641	2009
-63.2276, -3.1611	2008
-62.5744, -2.888	2015
-66.2995, -4.5965	2005-2006
-65.604, -4.0085	2015
-65.5602, -4.2885	1997-1998
-65.88371, -4.26384	2013
-61.0484, -2.9538	2017
-61.21955, -2.9354	1995

-64.379, -2.1375	2004-2006
-65.0808, -1.9018	2009-2011
-66.51897, -2.80437	1996
-56.4371, 1.252	2019
-59.113, -4.822	2004
-59.2527, -7.5132	2002-2003
-60.8658, -4.0965	2005
-57.8228, -5.7402	2005
-55.8349, -3.6298	2017
-52.942, -11.1509	2012
-58.9445, -0.7978	2013
-58.0444, -2.2661	2007
-61.20502, -2.21518	2001
-61.20502, -2.21518	2002-2003
-60.4921, -2.1428	2007
-61.61286, -0.64093	2019
-61.46795, -0.63504	1995
-61.1749, -1.7514	1994
-61.35325, -1.55977	2010-2013
-56.4458, -0.8913	2018
-60.70382, -2.54296	2014
-60.2861, -2.8878	2015
-60.41538, -2.67672	2016
-56.83937, -3.367	2017
-59.3013, -1.1196	2019
-56.3642, -1.1134	2011
-57.78923, -3.5598	1999
-58.7021, -1.6938	2001
-59.2186, -0.0446	2017
-58.7106, -0.4257	2008
-58.9853, -1.0443	2019
-62.052, -3.4237	2008
-61.9802, -3.6389	2008

Supplementary Table 3 The occurrence date of 38 windthrows

Latitude	Longitude	Year	Month	Date	Area of windthrows (hectares)
-2.93377	-75.28461	2011	9	3	1239.64227
-4.56275	-71.07156	2008	9	10	909.08325
-3.9108	-69.5837	2004	12	30	743.40522
-0.399	-69.9149	2009	9	3	609.61869
-0.4903	-70.146	2009	9	2	855.76545
-4.04714	-71.49935	2008	9	10	1145.95227
-3.0639	-71.7809	2004	9	17	479.1850587
-3.8458	-74.4379	2011	3	4	1467.62991
-2.4083	-71.5945	2004	9	6	305.8425
-4.9434	-70.9944	2008	9	9	593.98623
-3.75808	-74.25175	2017	7	30	192.1581
-1.76144	-71.16435	2017	8	21	126.39204
-2.46262	-70.10827	2010	9	26	703.89171
-2.68942	-70.9078	2010	10	1	156.93381
-1.135	-68.9531	2010	9	26	694.86417
-2.74979	-61.91331	2003	8	25	551.52522
-2.9963	-61.12781	2017	9	25	410.17194
-1.12337	-61.49781	2019	8	8	544.71456
-2.8039	-59.1245	2000	10	16	820.84653
-0.31402	-62.60639	2006	8	30	87.75279
-3.55848	-65.57446	2006	9	4	385.21017
-1.85828	-57.91189	2008	10	19	230.02164
-2.9454	-64.1962	2009	9	16	1029.95748
-1.8356	-66.7111	2018	9	8	91.29843
-3.6728	-65.3092	2008	8	11	430.38666
-2.888	-62.5744	2015	12	3	56.88
-4.0085	-65.604	2015	1	31	170.96823
-4.26384	-65.88371	2013	8	27	52.38
-2.9538	-61.0484	2017	9	28	58.2201
-4.822	-59.113	2004	9	16	1227.86505
-4.0965	-60.8658	2005	8	26	777.92823
-5.7402	-57.8228	2005	9	27	54.13302
-0.7978	-58.9445	2013	8	26	692.85276
-0.8913	-56.4458	2018	9	26	37.08
-2.54296	-60.70382	2014	8	11	62.19135
-2.8878	-60.2861	2015	11	13	65.38086

-2.67672	-60.41538	2016	8	4	89.1
-1.0443	-58.9853	2019	9	23	96.98184

Supplementary References

1. Seeley, J. T. & Romps, D. M. The effect of global warming on severe thunderstorms in the United States. *J. Clim.* **28**, 2443–2458 (2015).
2. Xin, Xiaoge; Wu, Tongwen; Shi, Xueli; Zhang, Fang; Li, Jianglong; Chu, Min; Liu, Qianxia; Yan, Jinghui; Ma, Qiang; Wei, M. BCC BCC-CSM2MR model output prepared for CMIP6 ScenarioMIP ssp585. *Earth System Grid Federation* https://doi.org/10.22033/ESGF/CMIP6.3050 (2019).
3. Swart, Neil Cameron; Cole, Jason N.S.; Kharin, Viatcheslav V.; Lazare, Mike; Scinocca, John F.; Gillett, Nathan P.; Anstey, James; Arora, Vivek; Christian, James R.; Jiao, Yanjun; Lee, Warren G.; Majaess, Fouad; Saenko, Oleg A.; Seiler, Christian; Seinen, M. CCCma CanESM5 model output prepared for CMIP6 ScenarioMIP ssp585. *Earth System Grid Federation.* https://doi.org/10.22033/ESGF/CMIP6.3696 (2019).
4. Li, L. CAS FGOALS-g3 model output prepared for CMIP6 ScenarioMIP ssp585. *Earth System Grid Federation* https://doi.org/10.22033/ESGF/CMIP6.3503 (2019).
5. Shiogama, Hideo; Abe, Manabu; Tatebe, H. MIROC MIROC6 model output prepared for CMIP6 ScenarioMIP ssp585. *Earth System Grid Federation* https://doi.org/10.22033/ESGF/CMIP6.5771le (2019).
6. Tachiiri, Kaoru; Abe, Manabu; Hajima, Tomohiro; Arakawa, Osamu; Suzuki, Tatsuo; Komuro, Yoshiki; Ogochi, Koji; Watanabe, Michio; Yamamoto, Akitomo; Tatebe, Hiroaki; Noguchi, Maki A.; Ohgaito, Rumi; Ito, Akinori; Yamazaki, Dai; Ito, Akihiko; Takata, Kumiko, M. MIROC MIROC-ES2L model output prepared for CMIP6 ScenarioMIP ssp585. *Earth System Grid Federation* https://doi.org/10.22033/ESGF/CMIP6.5770 (2019).
7. Kim, YoungHo; Noh, Yign; Kim, Dongmin; Lee, Myong-In; Lee, Ho Jin; Kim, Sang Yeob; Kim, D. KIOST KIOST-ESM model output prepared for CMIP6 ScenarioMIP ssp585. *Earth System Grid Federation* https://doi.org/10.22033/ESGF/CMIP6.11249 (2019).
8. Yukimoto, Seiji; Koshiro, Tsuyoshi; Kawai, Hideaki; Oshima, Naga; Yoshida, Kohei; Urakawa, Shogo; Tsujino, Hiroyuki; Deushi, Makoto; Tanaka, Taichu; Hosaka, Masahiro; Yoshimura, Hiromasa; Shindo, Eiki; Mizuta, Ryo; Ishii, Masayoshi; Obata, Atsushi; Adachi, Y. MRI MRI-ESM2.0 model output prepared for CMIP6 ScenarioMIP ssp585. *Earth System Grid Federation* https://doi.org/10.22033/ESGF/CMIP6.6929 (2019).
9. Dix, Martin; Bi, Doahua; Dobrohotoff, Peter; Fiedler, Russell; Harman, Ian; Law, Rachel; Mackallah, Chloe; Marsland, Simon; O'Farrell, Siobhan; Rashid, Harun; Srbinovsky, Jhan; Sullivan, Arnold; Trenham, Claire; Vohralik, Peter; Watterson, Ian; Williams, R. CSIRO-ARCCSS ACCESS-CM2 model output prepared for CMIP6 ScenarioMIP ssp585. *Earth System Grid Federation* https://doi.org/10.22033/ESGF/CMIP6.4332 (2019).
10. Wieners, Karl-Hermann; Giorgetta, Marco; Jungclaus, Johann; Reick, Christian; Esch, Monika; Bittner, Matthias; Gayler, Veronika; Haak, Helmuth; de Vrese, Philipp; Raddatz, Thomas; Mauritsen, Thorsten; von Storch, Jin-Song; Behrens, Jörg; Brovkin, Victor;, E. MPI-M MPI-ESM1.2-LR model output prepared for CMIP6 ScenarioMIP ssp585. *Earth System Grid Federation* https://doi.org/10.22033/ESGF/CMIP6.6705 (2019).
11. Voldoire, A. CNRM-CERFACS CNRM-CM6-1 model output prepared for CMIP6 ScenarioMIP ssp585. *Earth System Grid Federation* https://doi.org/10.22033/ESGF/CMIP6.4224 (2019).
